# Vitamin D Status and Its Role in First-Time and Recurrent Urinary Tract Infections in Children: A Case-Control Study

**DOI:** 10.3390/children8050419

**Published:** 2021-05-20

**Authors:** Carmen Muntean, Maria Săsăran

**Affiliations:** 1 Department of Paediatrics I, “George Emil Palade” University of Medicine, Pharmacy, Sciences and Technology of Târgu Mureș, Gheorghe Marinescu Street No. 38, 540142 Târgu Mureș, Romania; duicucarmen@yahoo.com; 2 Department of Paediatrics III, “George Emil Palade” University of Medicine, Pharmacy, Sciences and Technology of Târgu Mureș, Gheorghe Marinescu Street No. 38, 540142 Târgu Mureș, Romania

**Keywords:** vitamin D, urinary tract infections, children, recurrence, deficiency

## Abstract

Vitamin D has emerged as a key factor in innate immunity. Its involvement in the pathogenesis of urinary tract infections (UTIs) has gained a lot of attention recently. The objective of this study is to investigate the association between serum 25-hydroxyvitamin D (25(OH)D) levels and first-time or recurrent UTIs in children. A prospective, case-control study was conducted on 101 pediatric patients, who were divided into two groups: 59 patients with UTIs and 42 age-matched healthy controls. Serum 25(OH)D was determined in each child and expressed in ng/mL. Vitamin D presented significantly lower values in study group subjects than in healthy controls (*p* < 0.01). Moreover, a significantly higher prevalence of vitamin D insufficiency and deficiency was found in children with UTIs (*p* < 0.01). Patients with recurrent UTIs presented significantly lower levels of vitamin D than those with first-time UTIs (*p* = 0.04). Urinary tract abnormalities did not seem to exercise an additional effect upon vitamin D levels within the study group. In conclusion, first-time and recurrent UTIs are associated with lower vitamin D levels. Further studies are necessary to validate our findings, as well as future longitudinal research regarding efficacy of vitamin D supplementation in children with UTIs.

## 1. Introduction

Urinary tract infection (UTI) represents one of the most frequent bacterial infections in children [1]. *Escherichia coli* (*E. coli*) is the most often incriminated etiological agent, accounting for 80–90% of UTIs in children [2]. Potential evolution of UTIs towards serious complications such as sepsis and renal scarring, potentially responsible for permanent kidney damage, might be facilitated by a congenital malformation of the genitourinary tract [3].

Both bacteria and host factors play important roles in the development of UTIs [4]. Vitamin D has emerged as a major player in host innate immunity [5]. Considered an antimicrobial agent for a long time, vitamin D is also involved in urothelium protection, by stimulating the local production of antimicrobial peptides (AMP) [6]. Cathelicidin expression is stimulated by 1,25-dihydroxy vitamin D and protects the urinary tract from infection through induction of cytokine secretion [7]. An increase in β-defensin, another endogenous AMP, can also be noted with UTIs and is also modulated by 1,25-dihydroxy vitamin D [8]. Furthermore, vitamin D also helps in maintaining the integrity of the urothelium, through its receptor (vitamin D receptor-VDR), which is directly involved in regulating the function of epithelial cell junctions [9]. Uropathogenic *E. coli* acts directly on these epithelial cells, by downregulating claudin and occludin and thus disrupting this epithelial barrier [10].

Almost 99.9% of vitamin D circulates in the blood bound by specific and nonspecific proteins, namely vitamin D-binding protein (DBP) and albumin, respectively. According to a new hypothesis, only the unbound fraction (the free fraction) can cross the cell membrane and exert biological effects [11]. DBP binds vitamin D and its metabolites, but its production is not controlled by vitamin D itself, nor by its metabolic products [12].

The extent to which vitamin D insufficiency or deficiency can influence the development of UTIs is still controversial. Some authors have reported an increase in bacteriuria frequency only in patients with extremely low levels of vitamin D, of under 12.5 nmol/L [13]. Literature data on this matter is still limited in children. Various studies have reported associations between low serum vitamin D and increased risk of UTI [14,15], with one particular study concluding that serum levels of vitamin D lower than 25 nmol/L can constitute an independent risk factor for UTIs in pediatric populations [16]. However, some authors have reached intriguing conclusions, claiming that vitamin D supplementation can increase the risk of UTIs in children and infants [17,18]. Still, a small-scale study involving children of all ages supported the use of vitamin D in therapeutic doses (1000–5000 IU), for nine months, to efficiently reduce the incidence of recurring UTIs [19]. Furthermore, another study proved that therapeutic doses of vitamin D can prevent renal scaring in this category of patients [20].

The objective of this study was to investigate the relationship between serum 25-hydroxyvitamin D (25(OH)D) levels and first-time or recurrent UTIs in children.

## 2. Materials and Methods

A cross-sectional, case-control study was conducted on 101 Caucasian patients aged between 1 month and 12 years. The study enrolled 59 patients with UTIs, admitted in a Pediatric Tertiary Center from Romania and 42 healthy controls, who presented for routine check-ups to the pediatrician. The two groups were matched in terms of age, race, and social background. Patients with major heart defects, autoimmune disorders, diabetes mellitus, and digestive anomalies were excluded from the study group. Moreover, the control group did not include any patients with previously known chronic disorders, weight deficit, or obesity. An anamnesis was conducted in each of the patients’ parent/legal tutor regarding vitamin D supplementation. Each patient included in our study benefited from continuous vitamin D prophylaxis until the age of two (400 IU/day during the warm season and 800 IU/day during the cold season) and 800 IU/day during the cold season after the age of two.

Diagnosis of UTI was based upon symptoms, such as fever (≥38 °C), abdominal and lumbar pain, dysuria, pollakiuria, hematuria, inappetence and nausea, as well as urine analysis (leukocyturia, bacteria in the urinary sediment) and urine culture. Positivity of urine culture was defined as the presence of over 10^5^ colony forming units/mL of a single pathogen or over 10^4^ colony forming units/mL and clinical symptoms [21]. Urine sample collection was preceded by proper disinfection of the peri-urethral and genital regions. The clean-catch method of the urinary midstream was used for children with urinary continence and urinary catheters were used for infants and incontinent children. Considering the systemic symptoms and the modified urine culture, suggestive of upper UTI, an abdominal ultrasound (US) was conducted in each patient to search for possible anomalies of the urinary tract and kidneys, following the latest recommendations at the time of patient enrolment. In cases with US abnormalities voiding, cystourethrography was performed. Late Tc-99mDMSA (Technetium-99m 2,3-dimercaptosuccinic acid) was done in compliance with NICE (National Institute for Health and Care Excellence) in cases with atypical UTI, recurrent UTIs, and in those with evidence of renal parenchymal abnormalities at US or high-grade vesicoureteral reflux (VUR) discovered upon cystourethrography [22].

### 2.1. Laboratory Data

A complete blood count was performed in each patient belonging to the study group, with the help of an automated hematology analyzer (Cobas Integra 400 plus automated analyzer, Roche Diagnostics GmbH, Mannheim, Germany). The following serum biochemical parameters were also analyzed in these patients: urea, creatinine, calcium, magnesium, iron, phosphor, electrolytes (Na, K), total proteins and serum albumin, aspartate aminotransferase (AST), and alanine aminotransferase (ALT). Kidney function was assessed with the help of urea, creatinine levels, glomerular filtration rate (GFR), electrolytes, and urinalysis. C reactive protein (CRP) and erythrocyte sedimentation rate (ESR) were also evaluated. The spectrophotometric method was applied, with the help of an autoanalyzer (Cobas Integra 6000 plus automated analyzer, Roche Diagnostics GmbH, Mannheim, Germany). These investigations were conducted to assess the severity of the urinary tract infections, the inflammatory response, kidney and liver function. Serum 25(OH)D was determined for the entire study population with the help of chemiluminescence immunoassay and expressed in ng/mL. Insufficiency was defined for values between 20.1–29.9 ng/mL, whereas deficiency for levels lower or equal to 20 ng/mL.

### 2.2. Statistical Analysis

GraphPad PrismT software was used for statistical analysis. Descriptive statistics was useful for mean and median calculation of demographic and paraclinical data. Shapiro–Wilk normality test was applied for quantitative variables. For comparison of means and medians, Mann–Whitney test and unpaired *t*-test were used, depending on different parameter compliance to Gaussian distributions. Chi-square test was applied to assess the relationship between qualitative variables (sex, rural/urban background) and the risk of developing UTI. Assessment of mean vitamin D differences between multiple age groups was performed using the analysis of variance (ANOVA) test. Non-parametric Kruskal–Wallis test helped perform these multiple mean comparisons. Spearman analysis investigated the correlation between age, body temperature, hematological, biochemical parameters and serum 25(OH)D levels in the study group. Spearman’s correlation coefficients were calculated for each analysis. Multivariate analysis was used to assess cofounding factors involved in the development of UTIs (age, sex and vitamin D levels). *p* values were considered significant if they were under 0.05 (corresponding to a confidence interval of 95%).

### 2.3. Ethics

The research was conducted in accordance with the principles of the Helsinki declaration. An informed consent was obtained from the patients’ legal guardians prior to inclusion in the study. The study was approved by the ethics committee of “George Emil Palade” University of Medicine, Pharmacy, Sciences and Technology of Târgu Mureș (approval number 215/2019).

## 3. Results

### 3.1. Demographic Data and Vitamin D Levels

Comparison of demographic characteristics and vitamin D levels between the study and control group is depicted in Table 1. Mean age of patients included in the study was 3.95 ± 2.94 years for the study group and 3.25 ± 3.03 years for controls. No significant difference regarding age and urban/rural background was found between the two groups (*p* = 0.35 and *p* = 0.31, respectively). Still, distribution of age groups revealed a greater number of patients aged 2 years or more in the study group (*p* < 0.01). The male–female sex ratio of the study population was 1:1.4. Female sex proved to be a risk factor which can increase the likelihood of UTI development more than three times (OR = 3.034, *p* = 0.01). Vitamin D levels presented significantly lower values in study group subjects than in healthy controls (26.06 ± 14.25 SD versus 52.99 ± 23.16 SD, *p* < 0.01). As seen in Table 1, the majority of children (85.7%) included in the control group presented normal levels of 25(OH)D. None of them presented vitamin D deficiency. On the other hand, percentages of UTI subjects with normal, insufficient, or deficient levels of 25(OH)D were approximately equal. Comparison of these percentages between the two study groups revealed an important, higher prevalence of vitamin D insufficiency and deficiency in children with UTI (*p* < 0.01). Furthermore, comparison of mean vitamin D levels between females and males within the study group did not reveal any important differences (28.63 ± 15.28 SD versus 23.31 ± 11.34 SD, *p* = 0.22). Time of the year in which vitamin D dosing was conducted (warm or cold season) did not influence its serum levels significantly within our study population, despite slightly lower values during the cold season (24.07 ± 11.64 SD versus 28.38 ± 16.3 SD, *p* = 0.41).

### 3.2. Age Class Comparisons and Risk of UTI Recurrence in Relation to Vitamin D Levels

Correlations between age, body temperature, hematological, biochemical parameters, and serum 25(OH)D levels within the study group are presented in Table 2. Most of the analyzed parameters did not show any significant ascending or descending trends with 25(OH)D levels. However, an increase in calcium levels is positively correlated with vitamin D levels (r = 0.43, *p* < 0.01). This result is not surprising, as vitamin D promotes calcium absorption. On the other hand, higher ages seem to be associated with lower serum values of vitamin D (r = −0.52, *p* < 0.01).

As a result, a comparison of vitamin D levels and gender between the two study groups was performed, after dividing them into three age groups: <1 year of age, 1–2 years of age, and >2 years of age. Age of two was chosen as a milestone due to the continuous prophylaxis conducted until this age. Compellingly lower values of 25(OH)D and prevalence of vitamin D insufficiency and deficiency were found in the study group, in infants and in patients older than two years (Table 3). An interesting aspect was the absence of subjects with vitamin D deficiency in those younger than 1 year. Vitamin D levels were not significantly different for the age gap of 1–2 years (*p* = 0.16 and *p* = 0.58, respectively). Still, ANOVA test confirmed the descending trend of vitamin D levels with ageing in subjects with UTI (*p* < 0.01), but not in the control group (*p* = 0.08, Table 3). On the other hand, comparison of female/male ratio between the two study groups, divided within the three age groups revealed a positive, significant correlation of female sex and UTIs only for the age group of 1–2 years (*p* = 0.03), as seen in Table 3.

Assessment of 25(OH)D numbers in relation to recurrence of UTI is depicted in Table 3. Mean vitamin D levels of patients with first-time UTIs (29.65 ± 11.85 SD) were significantly higher than those of subjects with recurrent UTIs (24.82 ± 14.91 SD, *p* = 0.04).

Multivariate regression analysis was performed to evaluate the association between age, sex, and 25(OH)D levels and UTIs. Female sex represents a major risk factor for UTI, with an OR (odds ratio) of 4.12 (95% CI—confidence interval: 1.40–13.59), *p* = 0.02. Vitamin D levels are inversely associated with the risk of UTI, according to our study (OR = 0.92; 95% CI: 0.88–0.95, *p* < 0.01). Age was not associated with UTI risk (*p* = 0.22), although individual analysis of the three groups revealed a significant association with UTI risk for infants (OR = 4.33, 95 % CI: 0.8–29.44, *p* = 0.02) and children >2 years of age (OR = 2.15, CI:0.22–2.15, *p* = 0.01).

More than half of the patients (57.62%) included in the study also presented congenital abnormalities of the urinary tract. Half of these were represented by VUR, whereas the other half by other anomalies, such as ureteropelvic junction obstruction, vesicoureteral stenosis, or congenital/acquired solitary kidney. Still, an underlying malformation of the urinary tract does not seem to influence the levels of vitamin D within the study group (*p* = 0.85 for vesicoureteral reflux and *p* = 0.08 in cases with other abnormalities of the urinary tract, respectively).

In terms of UTI etiology, *E. coli* was the most common causative agent, accounting for 83.05% of cases. Other microbial agents were scarcely represented: Pseudomonas aeruginosa in two cases, Klebsiella pneumoniae in three subjects, whereas Enterococcus faecalis, Morganella morganii, Methicillin-resistant Staphylococcus aureus and Proteus mirabilis were only found in individual cases.

## 4. Discussion

Apart from its role in skeletal development, vitamin D has been investigated in relation to several types of infectious processes in children. Viral respiratory tract infections [23], tuberculosis [24], acute otitis media [25], as well as digestive tract infections [26] seem to be prevented to a certain extent by vitamin D supplementation. Up-to-date data also emphasized that inadequate vitamin D levels are associated with higher susceptibility and worse outcomes, as well as a an elevated (double) mortality risk in patients with SARS-COV2 infections [27].

The main DBP function is to control/adjust circulating free, as well as total levels of vitamin D metabolites. A minimal percent of 25(OH)D is unbound (0.03%), while the remaining is bound to DBP and albumin, respectively (85% and 15%). Additionally, less than 0.5% of total 1.25(OH)_2_D_3_ is unbound [11]. DBP or group specific component globulin (GC-globulin) is encoded by a 35 kb length gene and serves multiple functions (immune functions, transporter of vitamin D, dysregulation of the actin scavenging system, fatty acids binding/transport). DBP is produced mainly in the liver, its production being influenced by hormones (estrogen, glucocorticoids), drugs (dexamethasone), and cytokines (IL-6 and TGFβ). DBP gene, located on chromosome 4, is one of the most polymorphic genes known so far, with more than 120 variants and over 1200 polymorphisms [11]. Therefore, DBP levels are influenced by DBP alleles. Lower DBP levels are typically associated with Gc2 allele carriers [11]. According to Fu L et al., Gc2 variant is also associated with a better response to vitamin D supplementation [28]. Kidney disorders such as nephrotic syndrome, acute kidney injury, acute tubular necrosis, chronic kidney disease, tubular acidosis may influence DBP transport capacity from the glomerular filtrate to the renal tubules. Gross proteinuria may determine low DBP, as well as insufficient 25(OH)D levels [11]. A limitation of our study is that we did not measure DBP levels and we did not look for a genetic background. However, our study group excluded patients with those aforementioned kidney conditions, as well as cases of proteinuria, in order to rule out possible secondary causes of DBP deficiency.

A direct relationship between hypocalcemia and low vitamin D levels was found in our study. Intestinal calcium absorption is known to be optimal in the case of D vitamin sufficiency. In the case of vitamin D deficiency or insufficiency, compensatory mechanisms will determine an increase in parathormone (PTH) levels in order to maintain calcium levels. This makes PTH level another marker for vitamin D insufficiency, but its values tend to normalize after proper cholecalciferol supplementation [29]. Still, recent studies have showed that intact PTH levels were correlated with increase in free 25(OH)D, but not with total 25(OH)D levels [30].

The effect of vitamin D on the urothelium has also been intensely studied, in light of recent studies underlying its key role in innate immunity [31]. Most of the studies investigating vitamin D and its relation to UTIs, conducted on pediatric populations, have shown that an insufficient or deficient level of vitamin D represents a risk factor for these types of infections [17]. Moreover, a meta-analysis conducted on nine pediatric studies confirmed a direct link between vitamin D insufficiency and increased risk of UTI [32]. Our study supports this theory, as children with UTIs presented compellingly lower values of serum 25(OH)D than their healthy counterparts, as well as a higher prevalence of vitamin D insufficiency and deficiency. This hypothesis is however contradicted by some authors, who claim that vitamin D supplementation can lead to light nephrocalcinosis, which favors bacterial proliferation [18]. This mechanism, together with inhibition of the immune system caused by 25(OH)D supplements, especially with high doses, are claimed to be responsible for augmenting the UTI risk [33].

Vitamin D levels presented a descending trend with thriving in our study, but only in children diagnosed with UTI. This finding is inconsistent with other studies evolving around UTI in children so far, which have not found any significant correlation between age and vitamin D levels [15,34]. The children included in our study benefited from continuous vitamin D prophylaxis until the age of two. Our national guidelines recommend continuous prophylaxis until the age of 18 months, but most of the Romanian children receive continuous vitamin D supplementation until the age of two, as the ones included in our study [35]. Cold season prophylaxis is considered sufficient after this age, as long as sun exposure is sufficient [36]. Therefore, significant differences in vitamin D levels in our study sample after the age of two might be explained by lack of sufficient UV (ultraviolet) exposure. On the other hand, an interesting aspect was the absence of infants with vitamin D deficiency among our study sample. Maternal vitamin D levels are known to have a great impact on 25(OH)D concentration of human milk, with evidence showing that vitamin D supplementation during lactation can significantly improve infant vitamin D status [37]. Therefore, maternal vitamin D serum values might have also influenced the 25(OH)D levels in infants. Lack of vitamin D dosing in most of the mothers of patients included in our study was an impediment towards evaluating possible maternal–infant correlations.

A high prevalence of female patients was also noted in the study group. A more than three-fold increase in UTI risk has been found in relation to the female sex. As mean vitamin D levels did not differ significantly between the two sexes in the study group, this result suggests that female sex is an independent factor for the development of UTIs, regardless of 25(OH)D status. Contrarily to our findings, a study conducted by Tekin et al., which also reported a higher percentage of girls among patients with UTI, claimed that female sex also exhibited lower serum 25(OH)D values [38]. Multivariate analysis also confirmed that female sex represented a risk factor for UTIs, in conjunction with low 25(OH)D levels, similarly to another study evaluating the relationship between vitamin D deficiency and UTIs [34]. Still, sex has also been compared separately on age groups. The age group of 1–2 years was the only one in which female sex was positively associated with UTIs. Lack of significant differences between the two sexes in infants is not surprising and reported in other studies as well, but similar findings after the age of two might be explained by the small study sample, as UTIs are progressively more common in girls with age increase [39,40]. The small number of patients might have also impacted comparison of urban vs. rural background within our study. We identified a higher percentage of children coming from an urban background within the control group, but without statistical significance. UTIs have also been reported in other studies less frequently among children coming from urban areas [41,42]. On the other hand, conurbations seem to slightly increase the risk of UTIs among general populations, also in relation to risky sexual behaviors [41].

Vitamin D supplementation for prevention of recurrent UTIs has represented a topic of interest in many studies recently. A randomized, triple-masked control trial has been conducted to investigate the effect that vitamin D supplements of 1000 IU/day has upon prevention of recurrence of UTIs as opposed to placebo. Administration of oral vitamin D drops did not prove to bring a significant benefit in preventing recurrence of UTIs. However, the authors implied that the protective effect of vitamin D could be obtained after achieving normal or higher levels of serum 25(OH)D in the affected population. Thus, the role of vitamin D supplementation should be reconsidered in the context of higher intake or prolonged duration of administration, according to the authors [43]. In our study, we obtained incontestably lower values of serum 25(OH)D levels in patients with recurrent UTIs than in the ones with first-time UTIs. Therefore, a future follow-up study to evaluate the incidence of UTI in these patients after reaching normal serum values of 25(OH)D could be useful.

Similarly to other pediatric studies on the same subject [16,38], *E.coli* was the most frequently encountered etiological agent in our research as well. An in vitro study performed on mice demonstrated an association between uropathogenic *E. coli* and upregulation of VDR levels. Vitamin D deficient mice presented more invasive forms of UTIs, as vitamin D is mandatory for transcriptional activity of VDR [44]. Therefore, normal serum levels of vitamin D can protect against invasion of *E. coli* in the urinary tract.

A positive, innovative aspect of this study is the inclusion of patients with vesicoureteral reflux and other types of congenital urinary tract malformations within the study, as these anomalies are known to account for an important number of febrile UTIs. Patients with underlying congenital abnormalities did not present lower values than other children included in the study group, without an underlying malformation. Thus, it seems that these malformations have a role in the pathogenesis of urinary tract infection which is independent of vitamin D levels. Current literature data do not bring any information regarding the role of congenital urinary tract abnormalities on vitamin D levels, as pediatric studies on the same matter excluded these categories [16,38,45]. Still, this study included a relatively small number of patients, this being its major limitation. Therefore, testing the hypothesis of whether urinary tract malformations pose an additional effect upon vitamin D levels in patients with UTI would have been more appropriate on a larger study sample. Furthermore, most of the subjects included in the subjects were infants, toddlers, and pre-schoolers, as reflected by the mean age of both study groups. As a result, further studies on larger populations and involving more heterogeneous age groups are necessary to validate our findings.

## 5. Conclusions

Vitamin D presents significantly lower serum values in children with UTIs than in healthy controls, according to our study. A higher prevalence of vitamin D insufficiency and deficiency was also found in relation to UTIs. Vitamin D could also play an important role in the prevention of UTI recurrence, as proved by comparing serum levels in recurrent UTI patients to the ones of first-time UTI subjects. On the other hand, vitamin D status in children with UTIs was not additionally influenced by congenital abnormalities of the urinary tract in our study. Further studies performed at a wider scale are necessary to validate our findings, as well as future longitudinal research on vitamin D supplementation in children with UTIs.

## Figures and Tables

**Table 1:** Comparison of demographic characteristics and vitamin D levels between the study group and control group.

Parameter	^a^ Study Group (n = 59)	^a^ Control Group (n = 42)	*p* Value
Age (years)	3.95 ± 2.94	3.25 ± 3.03	0.35
Female sex (percentage)	33.66	13.87	0.01, OR = 3.034 (1.19–6.19)
Male sex (percentage) ^b^	24.75	27.72	
Urban background (percentage) ^b^	30.7	24.8	0.31, OR = 1.62 (0.73–3.63)
Rural background (percentage)	27.7	16.8	
25 (OH)D level (ng/mL; mean ± SD)	26.06 ± 14.25	52.99 ± 23.16	<0.01
Vit D-Normal values: >30 ng/mL (percentage)	30.5	85.7	<0.01
Vit D- Insufficiency: 20–30 ng/mL (percentage)	32.2	14.3	
Vit D- Deficiency: <20 ng/mL (percentage)	37.3	0	
Age <1 year (percentage)	16.8	24.2	<0.01
Age 1–2 years (percentage)	11.9	8.9	
Age >2 years (percentage)	30.3	7.9	

Legend: ^a^ mean value ± SD (standard deviation) for continuous variables and % for categorical variables; OR—odds ratio; ^b^ reference level for OR.

**Table 2:** Non-parametric Spearman correlations within the study group.

Variable	r	Serum 25(OH) D Levels-*p* Value
Age, years	−0.52	<0.01
Temperature, °C	0.2	0.12
ESR, mm/h	−0.01	0.89
Leukocytes/µL	0.03	0.81
Erithrocytes, ×10^6^/µL	−0.2	0.11
Hgb, g/dL	−0.17	0.17
Htc, %	−0.22	0.08
MEV, fL	−0.09	0.47
Platelets/µL	0.02	0.84
Neutrophils, %	−0.02	0.83
Lymphocytes, %	0.01	0.88
Monocytes, %	0.13	0.31
Calcium, mmol/L	0.43	<0.01
Magnesium, mmol/L	0.2	0.18
Iron, µmol/L	−0.02	0.85
Phosphorus, mmol/L	0.03	0.86
AST, U/L	0.25	0.06
ALT, U,l	0.15	0.25
Urea, mg/dL	−0.1	0.44
Creatinine, mg/dL	−0.21	0.09

Legend: r-Spearman’s correlation coefficient, ESR—erythrocyte sedimentation rate, Hgb–hemoglobin, Htc—hematocrit, MEV—mean erythrocyte volume, AST–aspartate aminotransferase, ALT–alanine aminotransferase.

**Table 3:** Comparison of age groups depending on 25(OH)D levels and female/male sex ratio.

Age (years)	Analyzed Parameter	^a^ Study Group (*n* = 59)	^a^ Control Group (*n* = 42)	*p* Value
<1 (*n* = 41)	25 (OH)D level (ng/mL; mean ±SD)	36.9 ± 21.8	58.3 ± 14.2	<0.01
	Vit D-Normal values: >30 ng/mL (percentage)	21.9	53.7	<0.01
	Vit D-Insufficiency: 20–30 ng/mL (percentage)	19.5	4.9	
	Female sex (percentage)	24.5	17	0.11, OR = 3.46 (1.57–8.37)
	Male sex (percentage) ^b^	17	41.5	
1–2 (*n* = 24)	25 (OH)D level (ng/mL; mean ±SD)	31.8 ± 14.9	49.4 ± 28.1	0.16
	Vit D-Normal values: >30 ng/mL (percentage)	33.3	37.5	0.58
	Vit D-Insufficiency: 20–30 ng/mL (percentage)	12.5	12.5	
	Vit D-Deficiency: <20 ng/mL (percentage)	4.2	0	
	Female sex (percentage)	34.3	18.7	0.03, OR = 2.8 (1.22–6.11)
	Male sex (percentage) ^b^	18.7	28.3	
>2 (*n* = 36)	25 (OH)D level (ng/mL)	17.8 ± 7.2	38.8 ± 8.6	<0.01
	Vit D-Normal values: >30 ng/mL (percentage)	2.8	27.8	<0.01
	Vit D-Insufficiency: 20–30 ng/mL (percentage)	13.9	5.5	
	Vit D-Deficiency: <20 ng/mL (percentage)	50	0	
	Female sex (percentage)	27.9	16.6	0.9, OR = 0.71 (0.31–1.64)
	Male sex (percentage) ^b^	38.9	16.6	
**Nonparametric ANNOVA (Kruskal–Wallis) test for comparison of the three age groups in terms of vitamin D levels**				
		Study group	<0.01	
		Control group	0.08	
Study group	**^a^ First time UTIs** **(*n* = 17)**	**^a^ Recurrent UTIs** **(*n* = 42)**		
25(OH)D levels	29.65 ± 11.85	24.82 ± 14.91	0.04	

Legend: ^a^ mean value ± SD (standard deviation) for continuous variables and % for categorical variables; OR–odds ratio; ^b^ reference level for OR.

## Data Availability

The datasets generated during and/or analyzed during the current study are available from the corresponding author on reasonable request.
